# Gridded material stocks in China based on geographical and geometric configurations of the built-environment

**DOI:** 10.1038/s41597-023-02830-8

**Published:** 2023-12-20

**Authors:** Jian Sun, Tao Wang, Nanxi Jiang, Zezhuang Liu, Xiaofeng Gao

**Affiliations:** 1 School of Public Policy and Administration, Chongqing University, 174 Shazheng Rd., Chongqing, 400044 China; 2 Key Laboratory of the Three Gorges Reservoir Region’s Eco-Environment, Ministry of Education, College of Environment and Ecology, Chongqing University, Chongqing, 400045 China; 3 College of Environmental Science and Engineering, Tongji University, 1239 Siping Rd., Shanghai, 200092 China; 4 UNEP-Tongji Institute of Environment for Sustainable Development, Tongji University, 1239 Siping Rd., Shanghai, 200092 China; 5 Institute of Carbon Neutrality, Tongji University, 1239 Siping Rd., Shanghai, 200092 China; 6 Key Laboratory of Drinking Water Science and Technology, Research Center for Eco-Environmental Sciences, Chinese Academy of Sciences, Beijing, 100085 China; 7 University of Chinese Academy of Sciences, Beijing, 100049 China

**Keywords:** Sustainability, Environmental impact

## Abstract

Material stocks have created alternative perspectives in many environmental and climate studies. Their significance nonetheless may be under-explored, partially due to scarcity of more precise, timely and higher-resolution information. To address this limitation, our present study developed a gridded material stocks dataset for China in Year 2000 and 2020, by examining the geographical distribution and geometric configurations of the human-made stock-containing environment. The stocks of twelve materials embodied in five end-use sectors and 104 products and constructions were assessed at a resolution of 1 × 1 km grid. Material intensity in each product or construction component was carefully evaluated and tagged with its geometric conformation. The gridded stocks aggregately are consistent with the stock estimation across 337 prefectures and municipalities. The reliability of our assessment was also validated by previous studies from national, regional, to grid levels. This gridded mapping of material stocks may offer insights for urban-rural disparities, urban mining opportunity, and climate and natural disaster resilience.

## Background & Summary

Human-made materials in use have attained an unprecedented scale on Earth, surpassing the total biomass of all living organism^1^. A staggering 800 gigatonnes (Gt) of materials stocked in various forms from buildings, infrastructure, to capital and consumer goods make a matter basis for human survival and development. Those material stocks as a pivotal role in shaping and potentially mitigating future resource and energy consumption has gained increasing prominence. Consequently, the systematic quantification and comprehensive evaluation of materials transformed and used within socioeconomic systems stands out as a fundamental prerequisite for the sustainable management of products and natural services^2^.

In contrast to the gradual and dispersed patterns of material stock accumulation observed in developed countries over centuries, China has witnessed a rapid pace in this process, occurring within a span shortest to just a few years. However, this accelerated and concentrated materialization has posed significant environmental challenges, accompanied by the accumulation of substantial secondary raw materials and urban mines within the built environment. Thus, gaining a comprehensive understanding of the spatial distribution of material stocks has become crucial for the effective implementation of resource management policies and offers valuable insights for the promotion of sustainable development patterns in urban entities. Previous research on material flows in China has evolved from a national-level accounting to provincial^3^, city-level^4^, and even grid-level analyses^5,6^. Nevertheless, the absence of standardized methodologies has hindered a precise quantification and localization of provincial-level and city-level material stock accounts down to the level of individual urban grids.

To address these limitations, we started from computing city- and prefectural-level material stocks using a bottom-up methodology. This involves considering 104 final products derived from 12 different materials spanning five end-use sectors in China. Subsequently, we employed multi-source remote sensing data and GIS databases to perform a detailed downscaling of each product. Based upon the geographical and geometric configurations of the human-made environment, the material-containing products and their position in the built environment were assessed. A comprehensive multi-year material stock spatial database for China could be eventually created. This database offers a granular breakdown by product and material types, with a spatial resolution of 1 km. The dataset emerges as a valuable resource for gaining insights into the spatiotemporal patterns in urban development, promoting sustainable urban growth, optimizing recycling and management of multiple waste streams, and supporting the transition towards a circular economy.

## Methods

### Estimation of material stocks

The stocks of 12 materials were assessed across five end-use sectors and 104 categories of products and constructions. The estimation was conducted at provincial and prefectural levels in Mainland China and covered both urban and rural areas. Data of product stocks primarily drew from the previous databases (e.g., Song *et al*.^3^ and Li *et al*.^4^) and were double checked with the regional statistics. The factors of material intensity per product were carefully reviewed and improved to make the stock estimation more consistent with historical flows of material production and use^7–16^ (Table S1). For buildings, we compared the total built-up area between prefectures and their belonging provinces. The areas per prefecture were modified to ensure alignment with provincial statistics. Regarding infrastructure, our contributions mainly focused on municipal utilities and water ports, wherein we expanded the varieties for municipal network facilities, and estimated the size of each port by the number of berths. Furthermore, industrial electricity consumption was employed to estimate the quantity of industrial machinery in each city, and the cumulative statistics for all cities were validated against China’s industrial machinery data calculated by Wang, *et al*.^17^ using a bottom-up approach. Multiple sources and details of our estimation can be found in the supplementary material.

### Downscaling of material stocks

**Fig. 1:**
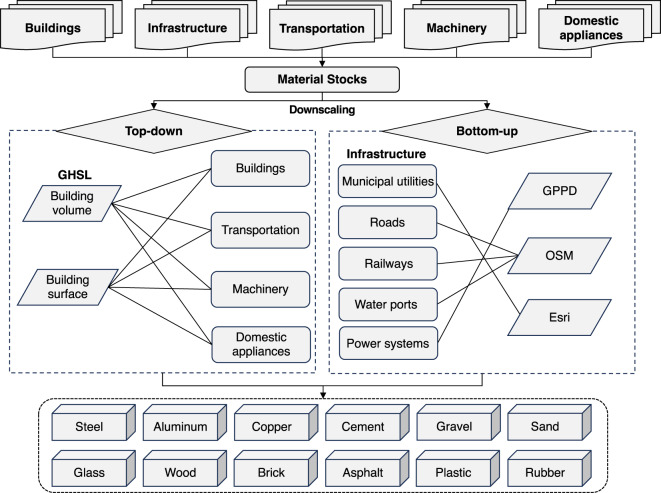
Schematic overview of the development of gridded material stocks dataset in China (GHSL: Global Human Settlement Layer, GPPD: Global Power Plant Database, OSM: OpenStreetMap dataset, Esri: Environmental Systems Research Institute dataset).

Two fundamental methods were employed for downscaling material stocks in this work: the bottom-up vs. the top-down approach. The top-down method relied on the linear relationship between material stocks and spatial raster distribution, and it was utilized for downscaling products within the buildings, transportation, domestic appliances, and machinery sectors. Conversely, the bottom-up approach directly mapped stocks to each grid cell by integrating the physical location and outlines of products within the infrastructure sector. Geographical data in this study were mainly derived from four sources: (1) The Global Human Settlement Layer (GHSL) data package^18^, (2) OpenStreetMap (OSM) dataset (https://planet.openstreetmap.org/planet/full-history/), (3) Global Power Plant Database^19^, and (4) Environmental Systems Research Institute (Esri) dataset (https://storymaps.arcgis.com/stories/011f587e84a246cf8f6c39ecbe785455). For detailed information regarding the calculation procedures, base maps, and subcategories of data, please refer to the supplementary materials. The schematic workflow of the research is illustrated in Fig. 1.

#### Buildings

Buildings in urban and rural areas were categorized into urban residential buildings (UR), urban non-residential buildings (UnR), rural residential buildings (RR), and rural non-residential buildings (RnR)^20–24^. Firstly, to demarcate material stocks between rural and urban material stocks, the GHSL settlement layers (version of GHS-SMOD R2022A)^25^, namely an open-data project providing global spatial information about the settlement classification over time, were employed to delineate urban and rural buildings geographically. Specifically, bands 30 to 21 were reclassified to represent regions corresponding to urban buildings, whereas bands 13 to 11 were designated to demarcate areas representing rural buildings. Secondly, the GHS built-up volume grids (version of GHS-BUILT-V R2022A)^26^ were utilized as a proxy for distribution of material stocks within buildings. These grids provided data on the total built-up volume (*TBV*) and the volume allocated to dominant non-residential (*NBV*) every five years, spanning from 1975 to 2020. The volume of residential (*RBV*) was consequently derived by subtracting the *NBV* from the *TBV*. Thirdly, taking into account the distinction between urban and rural areas, the downscaled base map was further subdivide into building volumes in urban areas (*TBV*_*u*_), total building volumes in rural areas (*TBV*_*r*_), volumes of urban residential buildings (*BV*_*ur*_), volumes of urban non-residential buildings (*BV*_*unr*_), volumes of rural residential buildings (*BV*_*rr*_), and volumes of rural non-residential buildings (*BV*_*rnr*_). Combined, the downscaling method implemented here was made by application of the formula:

$${\bar{S}}_{x,y}=\frac{{v}_{x,y}}{\sum _{x}{v}_{x,y}}\times {S}_{y}$$

where $\bar{s}$ is the total material stocks in grid *x* within a city *y*, and *v* is the corresponding pixel value of building volume, while *S*_*y*_ is the total material stocks within the city. The base map sources for all building categories are in Table S2.

#### Infrastructures

The infrastructure system primarily includes roads^27^, municipal utilities^28,29^, power systems^30^, railways^17^, water ports^31,32^. The downscaling approach employed parallels that of the building sector, entailing the establishment of a linear relationship between infrastructure data, whether in vector or raster format, and material stocks. This relationship facilitated the derivation of material stocks within each 1 km grid cell. In the case of vector data, the processing approach involved its rasterization into 1 km raster data.

#### Roads

We extracted the line layers containing the key labels: “motorway”, “trunk”, “primary”, “secondary”, “tertiary”, “bridge”, and “tunnel” from OSM dataset, which comprises crowd-sourced geospatial information, providing a detailed map of global features and locations. These extractions were performed separately to create the line configurations for “Expressways”, “Highways, Class I”, “Highways, Class II”, “Highways, Class III”, “Highways, Class IV”, “bridges”, and “tunnels” respectively. In order to acquire the road information for year 2000, we referred the road dataset provided by Baum-Snow, *et al*.^33^ through the Harvard Dataverse. We combined this dataset with provincial yearbooks, enabling us to retroactively estimate the roads at various administrative levels for each province. This approach was adopted to ensure the consistency between vector data and statistical information.

Furthermore, unlike the aforementioned records related to roads, “urban roads” and “urban bridges” were likely to be primarily relevant to urban residents. Although a relatively accurate layer to describe the internal urban roads and bridge was not found, considering the correlation between the distribution of urban roads and the land area occupied by buildings, the GHSL built-up surface grids (version of GHS-BUILT-S R2022A)^34^ were employed as the contour surface for downscaling. It can be further categorized into the following components: total building surface in urban areas (*TBS*_*u*_), total building surface in rural areas (*TBS*_*r*_), building surface of urban residential areas (*BS*_*ur*_), building surface of urban non-residential areas (*BS*_*unr*_), building surface of rural residential areas (*BS*_*rr*_), and building surface of rural non-residential areas (*BS*_*rnr*_). In this study, the *TBS*_*u*_ was employed as a proxy for urban roads and urban bridges (see Table S3 for details).

#### Municipal utilities

This research investigated six types of municipal utilities, encompassing water supply, sewage, heat supply, gas distribution, streetlamps, television and telecommunication networks. Given that a majority of municipal pipelines were typically buried underground, the *TBS*_*u*_ was selected as the gridded surface for mapping water supply pipelines, steam pipelines, hot-water pipelines, sewerage pipelines, and streetlamps. Furthermore, for the downscaling of “Gas supply pipelines” and “Liquefied petroleum gas pipelines”, we used the line layers with “oil” and “gas” from the Esri dataset. The estimation methodology for pipelines corresponding to year 2000 mirrored the approach employed for roads. The realm of communication networks included TV cables and landlines. Television cables and telecommunications cables shared similarities in connecting buildings and households. Consequently, the *TBV*_*u*_ and *TBV*_*r*_ were chosen as the gridded maps for downscaling. Table S4 presented an overview of municipal utilities downscaling.

#### Power systems

The power systems were composed of power generations, transformers and transmission wires. Firstly, the power generations cover wind power, hydropower, and coal-fired power. To compile geographic information for power generation in China, we gathered data from the Global Power Plant Database version of 1.3.0^19^. Secondly, each power station was geolocated and buffered to calculate their spatial coverage. These scattered polygons were subsequently rasterized and downscaled. Thirdly, regarding electricity transmission, the “ELECTRICITY” layer of Esri was used as a downscaling line geometry. Lastly, since substations are distributed around the city and have little correlation with building heights and attributes, the *TBS*_*u*_ distribution is the grid geometry as downscaled. Further details of can be found in Supplementary Table S5.

#### Railways and water ports

The railways comprised both conventional train lines and high-speed train routes, which can be further subdivided into various components such as bridges, tunnels, tracks, ties, turnouts, fences, electric communication, and signalling systems. A layer specifically detailing railway bridges or tunnels in China was not found. Instead, we estimated the length of railway bridges and tunnels by prefecture to reflect the varying densities of these structures across different areas. We extracted the railway features in line layers from OSM and rasterized them into 1 km grid. For water ports, the point layer containing the “ferry terminal” feature was employed for downscaling (see Table S6).

#### Transportation equipment

This study downscaled three categories of transport equipment, namely motor vehicles, railway rolling stocks, and vessels, which follow the geometric configurations of surfaces, lines and points respectively^35,36^. Specifically, although vehicles are mobile assets, a significant portion of their lifecycle is spent parked in parking lots and public spaces. Consequently, *TBS*_*u*_ and *TBS*_*r*_ were employed as the gridded surface for downscaling representation of urban and rural vehicles. Regarding railway vehicles, we initially computed rolling stocks by province, and subsequently downscaled these based on provincial calculations to the “railway” layer within each province, reflecting disparities between provinces. Similarly, the scattered points of the “ferry terminal” layer obtained by OSM was used to geolocate and downscale the motor vessels and barges.

#### Machinery

As fixed assets of enterprises or farmers, the downscaling of machinery follows the gridded surface of buildings. Considering that machines are typically situated in non-residential areas, and the standard height of industrial plants is usually only 5m-6m, the *BS*_*unr*_ and *BS*_*rnr*_ were employed for the downscaling of industrial machines and agricultural machines, respectively.

#### Domestic appliances

Many appliances for cleaning, cooking, and air conditioning utilize are fixed in buildings^37–40^. To capture regional disparities, we computed the quantity of domestic appliances across >330 prefectures in China. The lower ownership of domestic appliances per household caused by the high housing vacancy in prefectural cities in northern and western China had been considered. Then, given that the number of households is closely related to the volume of buildings, the *BV*_*ur*_ and *BV*_*rr*_ were selected as the gridded surface for downscaling the domestic appliances stocks between urban and rural areas, respectively (see Table S7).

## Data Records

A comprehensive set of material stocks estimates was produced for China in Year 2000 and 2020, containing twelve bulk materials (i.e., steel, aluminium, copper, cement, gravel, sand, glass, wood, brick, asphalt, plastic, and rubber) distributed across five sectors and 104 products. These GEOTIFF files provide detailed geographic information of material for each sector (i.e., buildings, infrastructure, transportation, machinery, and domestic appliances), at a spatial resolution of 1-km. The dataset is freely available through Figshare (10.6084/m9.figshare.24315517)^41^.

## Technical Validation

**Fig. 2:**
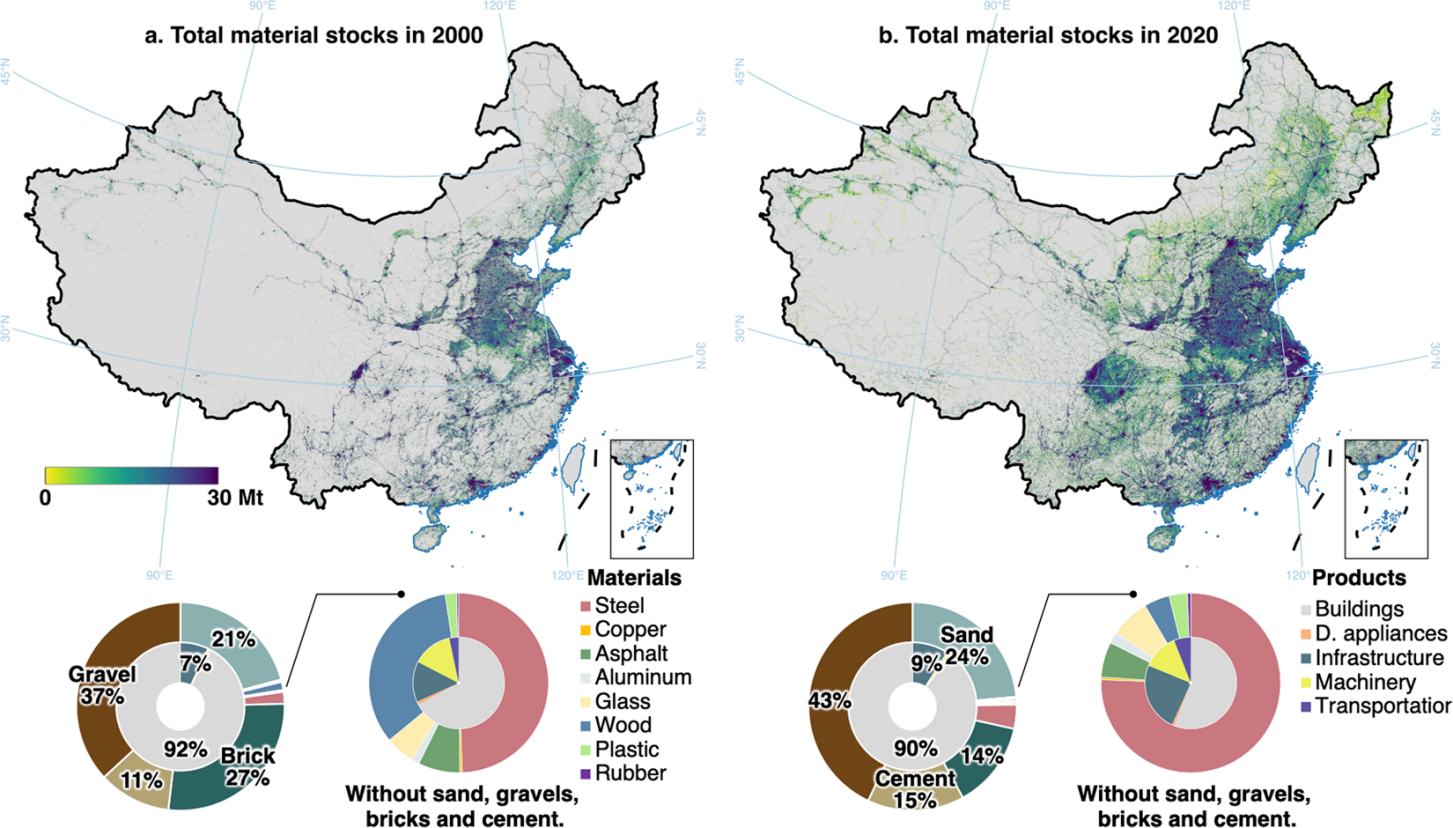
Total material stocks in China in 2000 (**a**) and 2020 (**b**), with a resolution of 1 × 1 km grid. The pie chart displays stock composition of materials in the outer circle and composition by products in the inner circle (D. appliance: Domestic appliance).

The material stocks atlas (Fig. 2) characterizes the stock distribution across China, broken down by materials and products. Over the course of two decades, the total material stocks witnessed a remarkable threefold increase from 73 Gt in 2000 to 219 Gt in 2020. Notably, the buildings and infrastructure played a predominant role, counting nearly 99% of the total material stocks. A different trend may emerge after excluding the bulk construction materials (i.e., sand, gravel, brick, and cement). The steel stocks in China expanded more than sixfold in the past two decades and accounted for a substantial 76% of the non-construction material stocks in 2020. Simultaneously, the massive use of plastics and rubber led to their stocks increasing some six and seven folds, respectively. The wood stocks, however, declined from 928 megatonnes (Mt) to 502 Mt, primarily attributed to the transformation in building structures and construction materials. Furthermore, the spatial distribution of material stocks revealed an evident expansion from eastern coast to inland China. The most significant expansion could be observed in various urban agglomerations, including the Beijing-Tianjin-Hebei region, the Yangtze River Delta, and the Sichuan-Chongqing basin. Additionally, there was a noted increase in stocks surrounding the central urban areas, illustrating clear signs of urban sprawl and robust infrastructure development.

**Fig. 3:**
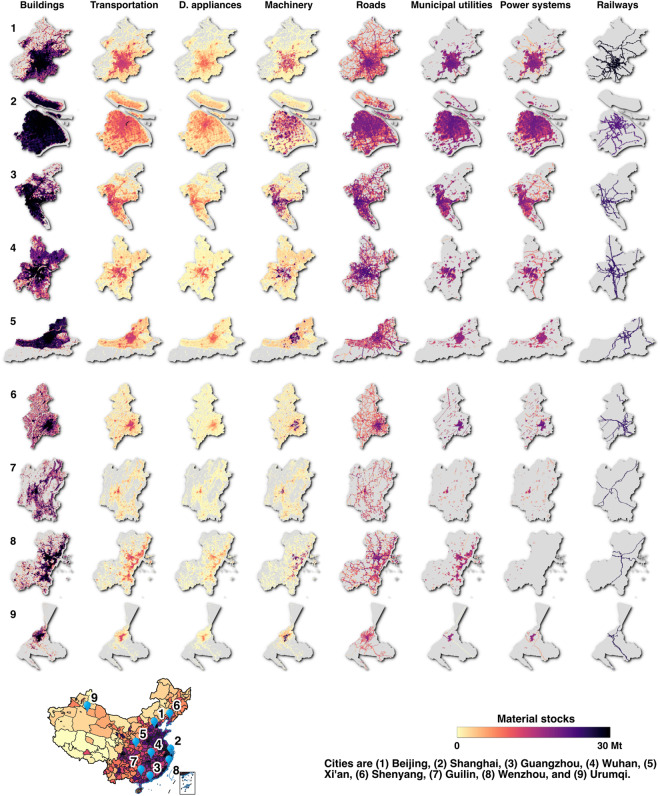
Gridded material stocks in representative cities by eight products (D. appliance: Domestic appliance).

**Fig. 4:**
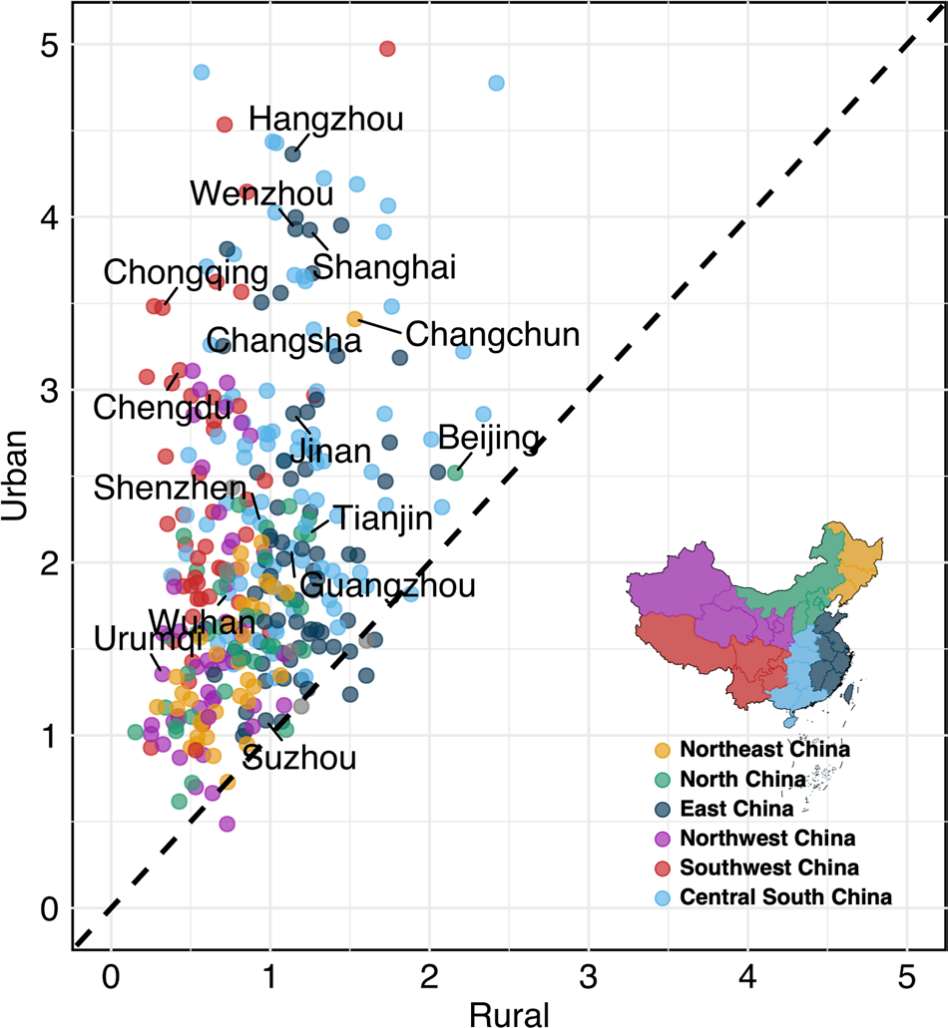
Times of 2020 to 2000 material stocks in urban and rural areas across Chinese prefectures.

Regionally, we selected nine representative cities to illustrate the spatial distribution patterns of material stocks across various products (Fig. 3). It is noteworthy that the building sector displayed an absolute dominance over material stocks in all these cities. However, the magnitude of the stocks exhibited notable disparities. For instance, in megacities such as Shanghai and Guangzhou, the material stocks density reached a remarkable nearly 0.7 Mt per square kilometers (Mt/km^2^). In contrast, the third-tier cities like Guilin and Urumqi exhibited considerably lower ratios, standing at only 0.05 and 0.1 Mt/km^2^, respectively. Larger cities also tended to demonstrate a more intensive infrastructure from roads, municipal utilities, to power systems. For example, the proportion of non-empty grid cells of roads to the total grid cells within the urban area in Shanghai extends to 71%, while it falls below 50% in big cities (e.g., 48% in Shenyang, 44% in Xi’an, and 39% in Wenzhou), and around 20% to smaller cities (e.g., 17% in Urumqi and 23% in Guilin). Nevertheless, the growth in material stocks per land within urban built-up areas had significantly outpaced that of rural areas. Across the majority of cities, urban built-up areas doubled their stock density, whereas rural areas have either maintained their existing status or displayed minor declines (i.e., stock density growth ≤1). Among these regions, the most substantial growth occurred in the Central-south China (Fig. 4), followed by the eastern and southwestern regions. As an example, in Hangzhou, the stock density within the urban built-up area impressively surged by a factor of 4.3 (from 0.3 Mt in 2000 to 1.4 Mt in 2020). In contrast, rural areas exhibited comparatively slower growth, increasing by a factor of 1.1 (from 0.07 Mt to 0.08 Mt). This discrepancy reflected a significant urban-rural disparity amid the backdrop of rapid urbanization.

**Fig. 5:**
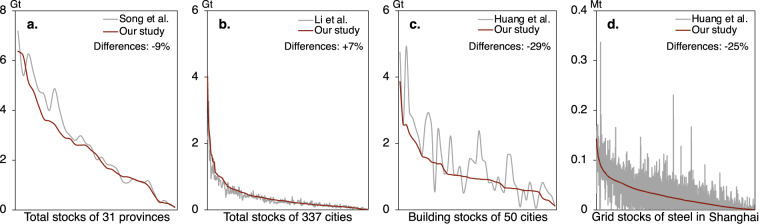
Validation of material stocks with existing datasets at provincial, city-, and grid-level.

The results were validated from national, provincial, and prefectural to grid levels. At the national scale, the country-level apparent material consumption was used to validate our estimates. Table 1 demonstrated a high degree of consistency between the different studies, with the differences no greater than 6%. At the provincial scale, our results were compared with the inventory of Song, *et al*.^3^. The absolute differences of total material stocks were less than 10%. The datasets of the Li, *et al*.^4^ and Huang, *et al*.^6^ were used for a validation at the prefectural level. It is clear that our results are more consistent with those of Li, *et al*.^4^ with average differences remaining at 7%. In contrast, the dataset developed by Huang, *et al*.^6^ which is gridded based on bottom-up machine learning estimates, displayed a greater divergence from our results, with a 29% difference in total materials in buildings at the prefectural level. Furthermore, we compared and assessed the gridded performance of our results from Huang, *et al*.^6^. A typical example is shown in Fig. 5d, which shows a comparison between the two datasets’ steel stocks of Shanghai at each pixel, where the major difference is 25%. It is evident that the overall trends of the two datasets are similar. The discrepancy can largely be attributed to the machine learning’s ability to capture a more extensive range of products at the surface. Upon examining the overall distribution pattern in the grid, we found that our gridded data in typical cities were fundamentally consistent with those in existing studies, providing further evidence of the practicability of our downscaling approach.

**Table 1:** Validation of material stocks between present and previous research.

Region	Reference	Year	Product	Material	Scope	Difference
China	Apparent consumption	2020	Total	Steel	National	−5%
China	Apparent consumption	2020	Total	Cement	National	+2%
China	Apparent consumption	2020	Total	Glass	National	+6%
China	Jiang *et al*.^45^	2000	Electronics	Plastic	National	−37%
China	Song *et al*.^3^	2000	Total	Total	Provincial	−9%
China	Wang *et al*.^17^	2000	Machinery	Steel	National	+9%
337 cities	Li *et al*.^4^	2020	Total	Total	City-level	+7%
50 cities	Huang *et al*.^6^	2020	Buildings	Total	City-level	−29%
Shanghai	Huang *et al*.^6^	2020	Buildings	Steel	Grid-level	−25%

## Usage Notes

The 12 materials from five end-use sectors encompass the majority of material usage by mass in social development and urban expansion. Material intensity on Figshare^41^ provides comprehensive information on the accounting scope. The data files are documented in GEOTIFF files, making them easily accessible for reading and processing using various programming and geographic analysis software, such as R language and QGIS. This dataset holds significant potential for monitoring the dynamics of socioeconomic development and human activities^42^, urbanization and construction^43^, and material usage across local^44^, regional, and national scales.

### Supplementary information

Supplementary Information

## Data Availability

The data gap is addressed using the R language and QGIS. The R code can be obtained from the open-access online dataset Figshare^41^.
