# Supplementary material for: Gridded material stocks in China based on geographical and geometric configurations of the built-environment
Source: Sci Data. 2023 Dec 20;10:915. doi: 10.1038/s41597-023-02830-8 (PMC10733388; doi:10.1038/s41597-023-02830-8)
Supplement: Supplementary file 1 — Supplementary Information [file 41597_2023_2830_MOESM1_ESM.pdf]

SUPPLEMENTARY INFORMATION

Gridded Material Stocks in China based on Geographical and Geometric Configurations of the Built-Environment

Jian Sun [1,2], Tao Wang [3,4,5], Nanxi Jiang [6,7], Zezhuang Liu [2], Xiaofeng Gao [2]

[1] School of Public Policy and Administration, Chongqing University, 174 Shazheng Rd., Chongqing, 400044, China

[2] Key Laboratory of the Three Gorges Reservoir Region’s Eco-Environment, Ministry of Education, College of Environment and Ecology, Chongqing University, Chongqing 400045, China

[3] College of Environmental Science and Engineering, Tongji University, 1239 Siping Rd., Shanghai, 200092, China

[4] UNEP-Tongji Institute of Environment for Sustainable Development, Tongji University, 1239 Siping Rd., Shanghai, 200092, China

[5] Institute of Carbon Neutrality, Tongji University, 1239 Siping Rd., Shanghai, 200092, China

[6] Key Laboratory of Drinking Water Science and Technology, Research Center for Eco-Environmental Sciences, Chinese Academy of Sciences, Beijing 100085, China

[7] University of Chinese Academy of Sciences, Beijing 100049, China

*Corresponding author: Tao Wang (a.t.wang@foxmail.com)

*Corresponding author: Xiaofeng Gao (gaoxiaofeng@cqu.edu.cn)

Table of contents

Table. S1 Material intensity for 12 materials……………………..............………..………………3

Table. S2 Overview of base map sources by building categories.…………………….........…….…4

Table. S3 Overview of base map sources by roads.……………………...........................……….5

Table. S4 Overview of base map sources by municipal utilities.…......…………………..….…..…6

Table. S5 Overview of base map sources by power systems..…7

Table. S6 Overview of base map sources by railways and water ports....…………......…….......…8

Table. S7 Overview of base map sources by transportation, machinery and domestic appliances...9

Table. S1 Material intensity for 12 materials.

End-use sectors	Products	Unit	Steel intensity[2,6,9]	Aluminum intensity[9,23]	Copper intensity[8,9,10]	Wood intensity	Cement intensity	Brick intensity	Gravel intenstiy	Sand intensity	Asphalt intensity	Glass intenstiy	Plastic intensity[4]	Rubber intensity
	Urban residential buildings[3,11]		3-65	0.15-2	0.15-0.35	40-1	130-400	760-250	470-1100	360-570	4-2	1.5-10	0.02-4	0.02-0.5
Buildings[12,13,14,24]	Rural residential buildings	kg/m^2	1-20	0.1-0.8	0.1-0.25	30-2	100-200	450-260	360-550	190-330	2-1	1-0	0.01-2	0.01-0.2
	Urban non-residential buildings[3,11]		5-80	0.15-2.8	0.15-0.45	35-1	145-540	800-300	550-1470	400-760	4.5-2.5	2-15	0.02-5	0.02-0.6
	Rural non-residential buildings		1-30	0.1-1.2	0.1-0.2	30-1	100-300	500-280	500-820	220-430	2.4-1.5	1-0	0.01-3	0.01-0.3
	Expressways[5]		190	1	0	—	115	—	365	440	397	—	—	—
	Highways, Class I[5]		2	1	0	—	110	—	349	450	294	—	—	—
	Highways, Class II[5]		1	1	0	—	68	—	216	390	191	—	—	—
	Highways, Class Ⅲ[5]		—	—	—	—	65	—	206	350	100	—	—	—
	Highways, Class Ⅳ[5]		—	—	—	—	59	—	187	330	66	—	—	—
	Urban roads[5]		0	1	0	—	59	—	187	330	66	—	—	—
	Highway bridges[5]		7	—	—	—	7	—	22	9	—	—	—	—
	Highway Tunnels[5]		5	—	—	—	4	—	12	5	—	—	—	—
	Urban bridges[5]		4	—	—	—	2	—	7	3	—	—	—	—
	Water supply pipelines-cast iron[16]	t/km	64	—	—	—	35	—	132	157	—	—	—	—
	Water supply pipelines-steel[16]		123	—	—	—	45	—	168	200	—	—	—	—
	Water supply pipelines-plastic[16]		—	—	—	—	20	—	76	91	—	—	5	—
	Water supply pipelines-others[16]		17	—	—	—	31	—	111	122	—	—	3	—
	Gas supply pipelines[29]		550	—	—	—	35	—	130	160	—	—	1	—
	Natural gas pipelines[29]		70	—	—	—	35	—	130	160	—	—	1	—
	Liquefied petroleum gas pipelines[29]		30	—	—	—	35	—	130	160	—	—	1	—
	Steam pipelines[29]		50	—	—	—	35	—	130	160	—	—	1	—
	Hot-water pipelines[29]		40	—	—	—	35	—	130	160	—	—	1	—
	Sewerage pipelines[16]		16	—	—	—	120	—	371	361	—	—	1	—
Infrastructure9[14,17]	Street lamps[29]	t/uint	0	—	0	—	—	—	—	—	—	—	—	—
	Hydropower stations[19]		150	0	3	—	1000	—	3170	1420	—	—	—	—
	Thermal power stations[19]	t/MW	80	0	1	—	150	—	476	213	—	—	—	—
	Wind power stations[19]		150	2	3	—	85	—	269	121	—	—	—	—
	500 kv voltage electricity transmission[19]		42	65	—	—	12	—	38	17	—	—	—	—
	330 kv voltage electricity transmission[19]		30	30	—	—	6	—	19	9	—	—	—	—
	220 kv voltage electricity transmission[19]	t/km	20	9	—	—	5	—	14	6	—	—	—	—
	110 kv voltage electricity transmission[19]		12	4	—	—	4	—	13	6	—	—	—	—
	35 kv voltage electricity transmission[19]		5	2	—	—	3	—	8	4	—	—	—	—
	500 kv voltage electricity transformation[19]		4	0	0	—	—	—	—	—	—	—	—	—
	330 kv voltage electricity transformation[19]		3	0	0	—	—	—	—	—	—	—	—	—
	220 kv voltage electricity transformation[19]	t/MVA	2	0	0	—	—	—	—	—	—	—	—	—
	110 kv voltage electricity transformation[19]		1	0	0	—	—	—	—	—	—	—	—	—
	35 kv voltage electricity transformation[19]		1	0	0	—	—	—	—	—	—	—	—	—
	Rural, cable TV users		—	—	0	—	—	—	—	—	—	—	—	—
	Urban, landline users	t/household	—	—	0	—	—	—	—	—	—	—	—	—
	Rural, landline users		—	—	0	—	—	—	—	—	—	—	—	—
	Water ports	t/berth	240	—	—	—	2265	—	4720	7880	—	—	—	—
	Railway	t/km	320	20	23	—	7249	—	32518	11038	—	—	—	—
	High-speed railway	t/km	596	24	26	—	8080	—	27849	13107	—	—	—	—
	Passenger cars, large[27]		7600	690	95	—	—	—	—	—	—	405	350	200
	Passenger cars, medium[27]		3200	270	50	—	—	—	—	—	—	135	300	155
	Passenger cars, small[27]		940	90	15	—	—	—	—	—	—	40	140	80
	Passenger cars, micro[27]		590	60	10	—	—	—	—	—	—	25	85	75
	Trucks, heavy		9200	375	110	—	—	—	—	—	—	490	300	790
	Trucks, medium	kg/unit	3700	250	55	—	—	—	—	—	—	150	260	395
	Trucks, light		1600	200	30	—	—	—	—	—	—	70	160	200
Transportation[18]	Trucks, micro		780	95	10	—	—	—	—	—	—	35	50	120
	Other vehicles[28]		2500	230	25	—	—	—	—	—	—	100	30	150
	Motorcycles		85	50	1	—	—	—	—	—	—	3.6	10	2
	Trailers		1500	260	—	—	—	—	—	—	—	—	60	90
	Freight cars		20	1.4	—	—	—	—	—	—	—	—	—	—
	Passenger coaches[28]	t/unit	23	1.6	0.26	—	—	—	—	—	—	0.26	3.4	—
	Locomotive		100	9	1.7	—	—	—	—	—	—	5.4	27	—
	Motor vessels	t/DWT	0.42	—	—	—	—	—	—	—	—	—	—	—
	Barges		0.38	—	—	—	—	—	—	—	—	—	—	—
	Large & medium tractors		3500	20	5	—	—	—	—	—	—	—	—	—
	Small tractors		1200	10	5	—	—	—	—	—	—	—	—	—
	Large & medium towing farm machinery		2000	—	—	—	—	—	—	—	—	—	—	—
	Small tractor towing farm machinery		800	—	—	—	—	—	—	—	—	—	—	—
	Threshing machine		2000	—	—	—	—	—	—	—	—	—	—	—
	Fishing machine		4000	1	2	—	—	—	—	—	—	—	—	—
Machinery[18]	Diesel engine	kg/unit	1200	—	—	—	—	—	—	—	—	—	—	—
	Electromotor		1200	—	2	—	—	—	—	—	—	—	—	—
	Drainage & irrigation machinery		400	5	2	—	—	—	—	—	—	—	—	—
	Harvesters		2000	5	2	—	—	—	—	—	—	—	—	—
	Transport power machinery		1000	5	5	—	—	—	—	—	—	—	—	—
	Pumps		500	1	2	—	—	—	—	—	—	—	—	—
	Fishing power boats		4000	2	2	—	—	—	—	—	—	—	—	—
	Industrial machinery[18]	t/wkwh	4-2.5	0.015-0.00825	0.008-0.00465									
	Urban, refrigerators		30	2.1	0.8	—	—	—	—	—	—	—	10	—
	Urban, washing machines		18	1.4	1.1	—	—	—	—	—	—	—	12	—
	Urban, fans[25]		0.8		0.2	—	—	—	—	—	—	—	1	—
	Urban, air conditioners		26	2.5	6.6	—	—	—	—	—	—	—	6	—
	Urban, water heaters		12	0.2	1.5	—	—	—	—	—	—	—	9	—
	Urban, combined sound		3.5	—	0.2	—	—	—	—	—	—	—	0.6	—
	Urban, digital video[25]		0.05	—	0.12	—	—	—	—	—	—	—	0.016	—
	Urban, camera[25]		0.05	—	0.05	—	—	—	—	—	—	—	0.04	—
	Urban, other musical instruments		4	—	0.2	—	—	—	—	—	—	—	0.12	—
	Urban, fitness equipment		10	—	—	—	—	—	—	—	—	—	0.6	—
	Urban, landline phones[25]		0.05	—	0.02	—	—	—	—	—	—	—	0.02	—
	Urban, mobile phones[25]		0.05	—	0.016	—	—	—	—	—	—	—	0.03	—
	Urban, pianos		40	—	0.2	—	—	—	—	—	—	—	8	—
	Urban, dish-washer		15	1.4	0.1	—	—	—	—	—	—	—	6	—
	Urban, disinfecting cabinet		15	—	0.1	—	—	—	—	—	—	—	15	—
Domestic appliances[1,14]	Urban, smoke absorbers[25]	kg/unit	20	—	0.1	—	—	—	—	—	—	—	8	—
	Urban, microwave ovens		8	0.5	0.8	—	—	—	—	—	—	—	2.5	—
	Urban, TV sets[15,22]		0.8	0.2	0.5	—	—	—	—	—	—	—	5	—
	Urban, computers[22]		1.5	0.5	0.5	—	—	—	—	—	—	—	0.35	—
	Urban, bicycles		8	3	0	—	—	—	—	—	—	—	1.5	—
	Urban, sewing machines		20	—	0	—	—	—	—	—	—	—	7	—
	Rural, refrigerators		30	2.1	0.8	—	—	—	—	—	—	—	10	—
	Rural, washing machines		18	1.4	1.1	—	—	—	—	—	—	—	12	—
	Rural, microwave ovens		8	0.5	0.8	—	—	—	—	—	—	—	2.5	—
	Rural, air conditioners		26	2.5	6.6	—	—	—	—	—	—	—	6	—
	Rural, smoke absorbers		20	—	0.1	—	—	—	—	—	—	—	8	—
	Rural, water heaters		12	0.2	1.5	—	—	—	—	—	—	—	9	—
	Rural, electric fans[25]		0.8	—	0.2	—	—	—	—	—	—	—	1	—
	Rural, computers[22]		1.5	0.5	0.5	—	—	—	—	—	—	—	0.35	—
	Rural, TV sets[15]		0.8	0.2	0.5	—	—	—	—	—	—	—	5	—
	Rural, bicycles		8	3	—	—	—	—	—	—	—	—	1.5	—
	Rural, sewing machines		20	—	—	—	—	—	—	—	—	—	7	—

Reference

1 Liu, Y., Song, L., Wang, W., Jian, X. & Chen, W.-Q. Developing a GIS-based model to quantify spatiotemporal pattern of home appliances and e-waste generation—A case study in Xiamen, China. Waste Management 137, 150-157, doi:https://doi.org/10.1016/j.wasman.2021.10.039 (2022).
2 Song, L., Wang, P., Xiang, K. & Chen, W.-Q. Regional disparities in decoupling economic growth and steel stocks: Forty years of provincial evidence in China. Journal of Environmental Management 271, 111035, doi:https://doi.org/10.1016/j.jenvman.2020.111035 (2020).
3 Yang, D. et al. Urban buildings material intensity in China from 1949 to 2015. Resources, Conservation and Recycling 159, 104824, doi:https://doi.org/10.1016/j.resconrec.2020.104824 (2020).
4 Jiang, X. et al. Assessment of Plastic Stocks and Flows in China: 1978-2017. Resources, Conservation and Recycling 161, 104969, doi:https://doi.org/10.1016/j.resconrec.2020.104969 (2020).
5 Guo, Z., Shi, H., Zhang, P., Chi, Y. & Feng, A. Material metabolism and lifecycle impact assessment towards sustainable resource management: A case study of the highway infrastructural system in Shandong Peninsula, China. Journal of Cleaner Production 153, 195-208, doi:https://doi.org/10.1016/j.jclepro.2017.03.194 (2017).
6 Song, L. et al. Mapping provincial steel stocks and flows in China: 1978–2050. Journal of Cleaner Production 262, 121393, doi:https://doi.org/10.1016/j.jclepro.2020.121393 (2020).
7 Cao, Z., Shen, L., Løvik, A. N., Müller, D. B. & Liu, G. Elaborating the History of Our Cementing Societies: An in-Use Stock Perspective. Environmental Science & Technology 51, 11468-11475, doi:https://doi.org/10.1021/acs.est.7b03077 (2017).
8 Zhang, L., Yang, J., Cai, Z. & Yuan, Z. Understanding the Spatial and Temporal Patterns of Copper In-Use Stocks in China. Environmental Science & Technology 49, 6430-6437, doi:https://doi.org/10.1021/acs.est.5b00917 (2015).
9 Liu, Q. et al. Product and Metal Stocks Accumulation of China’s Megacities: Patterns, Drivers, and Implications. Environmental Science & Technology 53, 4128-4139, doi:https://doi.org/10.1021/acs.est.9b00387 (2019).
10 Zhang, L., Cai, Z., Yang, J., Chen, Y. & Yuan, Z. Quantification and spatial characterization of in-use copper stocks in Shanghai. Resources, Conservation and Recycling 93, 134-143, doi:https://doi.org/10.1016/j.resconrec.2014.10.010 (2014).
11 Yang, D. et al. Spatial–temporal dynamics of the built environment toward sustainability: A material stock and flow analysis in Chinese new and old urban areas. Journal of Industrial Ecology 27, doi:https://doi.org/10.1111/jiec.13335 (2022).
12 Zhang, R. et al. What matters most to the material intensity coefficient of buildings? Random forest-based evidence from China. Journal of Industrial Ecology 26, 1809-1823, doi:https://doi.org/10.1111/jiec.13332 (2022).
13 Zhang, L., Lu, Q., Yuan, Z., Jiang, S. & Wu, H. A bottom-up modeling of metabolism of the residential building system in China toward 2050. Journal of Industrial Ecology 27, doi:https://doi.org/10.1111/jiec.13382 (2023).
14 Fu, C., Fath, B. D., Daigo, I., Zhang, Y. & Deng, T. Tracking urban metabolism flows through the lifecycle of buildings, infrastructure, and durable goods at material, product, and sector levels. Journal of Cleaner Production 336, 130402, doi:https://doi.org/10.1016/j.jclepro.2022.130402 (2022).
15 Wang, M., You, X., Li, X. & Liu, G. Watch more, waste more? A stock-driven dynamic material flow analysis of metals and plastics in TV sets in China. Journal of Cleaner Production 187, 730-739, doi:https://doi.org/10.1016/j.jclepro.2018.03.243 (2018).
16 Wang, T., Shi, F., Zhang, Q., Qian, X. & Hashimoto, S. Exploring material stock efficiency of municipal water and sewage infrastructures in China. Journal of Cleaner Production 181, 498-507, doi:https://doi.org/10.1016/j.jclepro.2018.01.253 (2018).
17 Shi, F. et al. Toward a Low Carbon–Dematerialization Society. Journal of Industrial Ecology 16, 493-505, doi:https://doi.org/10.1111/j.1530-9290.2012.00523.x (2012).
18 Wang, T., Müller, D. & Hashimoto, S. The Ferrous Find: Counting Iron and Steel Stocks in China's Economy. Journal of Industrial Ecology 19, n/a-n/a, doi:https://doi.org/10.1111/jiec.12319 (2015).
19 Li, F., Ye, Z., Xiao, X., Xu, J. & Liu, G. Material stocks and flows of power infrastructure development in China. Resources, Conservation and Recycling 160, 104906, doi:https://doi.org/10.1016/j.resconrec.2020.104906 (2020).
20 Pauliuk, S., Wang, T. & Müller, D. Steel all over the world: Estimating in-use stocks of iron for 200 countries. Resources Conservation and Recycling 71, 22–30, doi:https://doi.org/10.1016/j.resconrec.2012.11.008 (2013).
21 Zhang, Y. et al. Copper in-use stocks accounting at the sub-national level in China. Resources Conservation and Recycling 147, 49-60, doi:https://doi.org/10.1016/j.resconrec.2019.04.010 (2019).
22 Habuer, Nakatani, J. & Moriguchi, Y. Resource-availability scenario analysis for formal and informal recycling of end-of-life electrical and electronic equipment in China. Journal of Material Cycles and Waste Management 19, 599-611, doi:https://doi.org/10.1007/s10163-015-0452-1 (2017).
23 Wang, J. & Graedel, T. Aluminum in-use stocks in China: A bottom-up study. Journal of Material Cycles and Waste Management 12, 66-82, doi:https://doi.org/10.1007/s10163-009-0271-3 (2010).
24 Song, L. et al. China material stocks and flows account for 1978–2018. Scientific Data 8, 303, doi:https://doi.org/10.1038/s41597-021-01075-7 (2021).
25 Widmer, R., Oswald-Krapf, H., Sinha-Khetriwal, D., Schnellmann, M. & Böni, H. Global perspectives on e-waste. Environmental Impact Assessment Review 25, 436-458, doi:https://doi.org/10.1016/j.eiar.2005.04.001 (2005).
26 Sun, N. et al. Material Flow analysis of plastics from provincial household appliances in China: 1978–2016. Waste Management 153, 156-166 (2017).https://doi.org/10.1016/j.wasman.2022.08.031
27 Schmidt, W.-P. et al. Life cycle assessment of lightweight and end-of-life scenarios for generic compact class passenger vehicles. The International Journal of Life Cycle Assessment 9, 405-416, doi:https://doi.org/10.1007/BF02979084 (2004).
28 Castro, M. B. G., Remmerswaal, J. & Reuter, M. Life cycle impact assessment of the average passenger vehicle in the Netherlands. International Journal of Life Cycle Assessment 8, 297-304, doi:https://doi.org/10.1007/BF02978922 (2003).
29 Sun, J., Wang, T., Lu, S., Gao, X. & Du, H. Leverage of resource efficiency over environmental emissions: Case of a megacity in China. *The Science of the total environment* **858**, 159514, doi:https://doi.org/10.1016/j.scitotenv.2022.159514 (2022).
30 Yokoi, R., Nakatani, J., Hatayama, H. & Moriguchi, Y. Dynamic analysis of in-use copper stocks by the final product and end-use sector in Japan with implication for future demand forecasts. Resources, Conservation and Recycling 180, 106153, doi:https://doi.org/10.1016/j.resconrec.2022.106153 (2022).

Table. S2 Overview of base map sources by building categories.

Sectors	Categories	Base maps	Sources	Geometry
Buildings	Urban residential	BV_{ur}	GHSL	Surface
	Rural residential	BV_{rr}	GHSL	Surface
	Urban non-residential	BV_{unr}	GHSL	Surface
	Rural non-residential	BV_{rnr}	GHSL	Surface

Table. S3 Overview of base map sources by roads.

Sectors	Categories	Base maps	Sources	Geometry
Roads	Expressways	Motorway	OSM	Linear
	Highways, Class I	Trunk	OSM	Linear
	Highways, Class II	Primary	OSM	Linear
	Highways, Class Ⅲ	Secondary	OSM	Linear
	Highways, Class Ⅳ	Tertiary	OSM	Linear
	Bridges	Bridges	OSM	Linear
	Tunnels	Tunnels	OSM	Linear
	Urban roads	TBS_u	GHSL	Surface
	Urban bridges	TBS_u	GHSL	Surface

Table. S4 Overview of base map sources by municipal utilities.

Sectors	Categories	Base maps	Sources	Geometry
Municipal utilities	Water supply pipelines	TBS_u	GHSL	Surface
	Gas supply pipelines	Gas	Esri	Linear
	Liquefied petroleum gas Pipelines	Oil	Esri	Linear
	Natural gas pipelines	TBS_u	GHSL	Surface
	Steam pipelines	TBS_u	GHSL	Surface
	Hot-water pipelines	TBS_u	GHSL	Surface
	Sewerage pipelines	TBS_u	GHSL	Surface
	Streetlamps	TBS_u	GHSL	Surface
	Urban TV cables	TBS_u	GHSL	Surface
	Rural TV cables	TBV_r	GHSL	Surface
	Urban landlines	TBV_u	GHSL	Surface
	Rural landlines	TBV_r	GHSL	Surface

Table. S5 Overview of base map sources by power systems.

Sectors	Categories	Base maps	Sources	Geometry
Power systems	Hydropower stations	Hydro	Global Power Plant	Point
	Thermal power stations	Thermal	Global Power Plant	Point
	Wind power stations	Wind	Global Power Plant	Point
	Electricity transmission	Electricity	Esri	Linear
	Electricity transformation	TBS_u	GHSL	Surface

Table. S6 Overview of base map sources by railways and water ports.

Sectors	Categories	Base maps	Sources	Geometry
Railways and water ports	Railways	Railway	OSM	Linear
	Water ports	Ferry terminal	OSM	Point

Table. S7 Overview of base map sources by transportation, machinery and domestic appliances.

Sectors	Categories	Base maps	Sources	Geometry
Transportation	Urban motor vehicles	TBS_u	GHSL	Surface
	Rural motor vehicles	TBS_r	GHSL	Surface
	Railway rolling stocks	Railway	OSM	Linear
	Vessels	Ferry terminal	OSM	Point
Machinery	Agriculture	BS_{rnr}	GHSL	Surface
	Industrial	BS_{unr}	GHSL	Surface
Domestic appliances	Urban domestic appliances	BV_{ur}	GHSL	Surface
	Rural domestic appliances	BV_{rr}	GHSL	Surface
